# CRISPR Diversity in *E*. *coli* Isolates from Australian Animals, Humans and Environmental Waters

**DOI:** 10.1371/journal.pone.0124090

**Published:** 2015-05-06

**Authors:** Maxim S. Sheludchenko, Flavia Huygens, Helen Stratton, Megan Hargreaves

**Affiliations:** 1 Smart Water Research Centre, Griffith University, Southport, Queensland, Australia; 2 University of the Sunshine Coast, Sippy Downs, Queensland, Australia; 3 Institute of Health and Biomedical Innovation, Queensland University of Technology, Brisbane, Queensland, Australia; St. Petersburg Pasteur Institute, RUSSIAN FEDERATION

## Abstract

Seventy four SNP genotypes and 54 *E*. *coli* genomes from kangaroo, Tasmanian devil, reptile, cattle, dog, horse, duck, bird, fish, rodent, human and environmental water sources were screened for the presence of the CRISPR 2.1 loci flanked by *cas2* and *iap* genes. CRISPR 2.1 regions were found in 49% of the strains analysed. The majority of human *E*. *coli* isolates lacked the CRISPR 2.1 locus. We described 76 CRISPR 2.1 positive isolates originating from Australian animals and humans, which contained a total of 764 spacer sequences. CRISPR arrays demonstrated a long history of phage attacks especially in isolates from birds (up to 40 spacers). The most prevalent spacer (1.6%) was an ancient spacer found mainly in human, horse, duck, rodent, reptile and environmental water sources. The sequence of this spacer matched the intestinal P7 phage and the pO111 plasmid of *E*. *coli*.

## Introduction

Current water quality research is predominantly focused on identifying sources of faecal contamination in environmental water. However, despite many advances, utilizing many different technologies, tracing faecal water contamination remains problematic. In this work we investigated diversity of CRISPR in isolates from animals, humans and environmental waters and the potential of using outcome of that studies for microbial source tracking. The CRISPR system is a recently discovered immune-like defence system of bacteria and archaea against phages and plasmids [1, 2]. Mechanisms of this defence system have been well studied, however CRISPR adaptation remains poorly understood [3, 4]. The system is based on the retention of specific sequences (proto-spacers) from mobile genetic elements during the first infection/integration. These are harboured (as spacers) within so-called CRISPR loci [5]. These spacers are transcribed with handles comprised of the repeats into short interfering CRISPR RNA molecules (crRNA), and are subsequently used to interfere (commonly referred to as ‘silencing’) with the known, or recognised, foreign DNA or RNA to cleave foreign nucleic acid in order to protect the cell [6]. Intriguingly, whilst about 96% of archaea contain CRISPR genes, only about 45% of bacteria have them [7]. Nevertheless, the diversity of CRISPR arrays have been successfully utilized in bacterial genotyping mainly used for spoligotyping in *Mycobacterium tuberculosis* which is based on spacer variation detection [8].

CRISPR are characterised by multiple palindromic repeats of 30–60 bp length, with spacers of approximately the same size located between them (over 300 elements per CRISPR locus) [9]. The spacer located next to the first repeat, with respect to the AT-rich leader sequence (5), is considered to be the newest and likely targets the most recent plasmid and phage challenges. Importantly, the CRISPR associated (*cas*) genes that encode genes involved in the processing of crRNA and recognition of foreign DNA, vary in structure and function depending on the bacterial species in question [10]. For example in *E*. *coli* type systems Cas 1 and Cas 2 are shown to be essential for the acquisition of new spacers from phages [11, 12]. To our knowledge the only known spacer that matches well characterized enterobacteria phages from Genbank is P1 from *E*. *coli* [13]. Additionally, the role of old spacers, the last spacers in regards to leader location, is uncertain because the direct repeats which surround them are usually degenerated [14].

Currently, knowledge of CRISPR genes has been applied to: (i) typing activities such as spoligotyping in *M*. *tuberculosis* (8), *Corynebacterium diphtheriae* [15], subtyping of *Yersinia pestis* [16], *Campylobacter jejuni* [17] and *Salmonella enterica* [18]; (ii) industrial activities such as engineering of dairy starter cultures to be resistant to phage attack and (iii) versatile tool for genome engineering [19, 20].

Importantly, the spacers in bacterial genomes are considered to be potential sub-typing markers for both host cells and their viruses [21]. By using knowledge of the spacer sequences and their position in the pattern, the bacteriophage sequences that parasitised the host in the past could be identified.

CRISPR system in *E*. *coli* contains two subtypes: I-E and I-F [22]. CRISPR I-E type consists of three cassettes: CRISPR 2.1, CRISPR 2.3 and CRISPR 2.2 loci [21]. Diversity in CRISPR 2.1 and CRISPR I-F is highest suggesting that these loci are intensively involved in cellular defence. The CRISPR 2.1 locus in *E*. *coli* is considered to be the result of the most recent attack events and therefore has the longest and most informative loci [23]. For this reason, and the lack of CRISPR I-F in the majority of *E*. *coli* genotypes [21], CRISPR 2.1 was selected as an appropriate locus for *E*. *coli* strain differentiation and as a potential tool for microbial source tracking. To our knowledge the macro-host specificity of *E*. *coli* spacers and the potential of these separately harboured spacers, remains to be investigated.

Recently, we reported development of a Single-Nucleotide Polymorphism Real-Time (SNP) genotyping method [24] for the purpose of determining host-specificity of *E*. *coli* isolated from water. Eight human-specific SNP profiles were identified and majority of them detected in environmental water samples [25]. However, more than half *E*. *coli* SNP profiles detected were unresolved because they were originated from ‘mixed’ sources being either human and/or animal isolates. For instance, SNP profile 18 included duck, horse, cattle and human *E*. *coli* isolates. From this work, we postulated that CRISPR genes might also prove useful in this context, to increase the discriminatory power of SNP–typing method developed previously.

A combination of SNP profiling based on conservative housekeeping genes and highly-variable areas of CRISPR loci was shown previously to be useful for the characterisation of a clinical population of *Campylobacter jejuni* [17]. The authors identified the specific clonal complexes using CRISPR loci, which dramatically increased the discrimination power of highly conservative SNP profiles of *C*. *jejuni*. Combination studies using CRISPR loci for dividing SNP profiles of *E*. *coli* isolates demonstrated a high level of differentiation, and should be at least as useful as was found in the case of the less variable clinical *C*. *jejuni* population.

The aim of this study was to identify spacer diversity within a collection of *E*. *coli* isolates. We successfully sequenced 45 individual CRISPR alleles from a set of SNP profiles detected in a diverse number of *E*. *coli* isolates from Australian animals (indigenous and introduced), water samples (lake and rivers), commensal (faeces), and clinical (urine and blood) human samples. The analysis was also applied to *in silico* CRISPR sequences from Australian carnivorous marsupials, fish, Tasmanian devil, environmental water and human faeces (clinical and commensal) in an effort to assess, and potentially extend, the methods utility for such analyses.

## Materials and Methods

### *E*. *coli* isolate collection

*E*. *coli* isolates (N = 185), from a range of sources (animals, water and human) [24] and previously characterized using SNP analysis [24], were selected for CRISPR screening (see Table 1). Cultures were incubated overnight at 37°C in 5 mL nutrient broth (Oxoid, UK) followed by DNA extraction.

**Table 1:** Presence of CRISPR2.1 regions in South East Queensland isolates and Australian *in silico* strains (shown in italics).

Source	Isolate/Strain code number^a^	CRISPR 2.1 Present (Y/N)
**Human (N = 30)**	hu7; hu12; hu15; hu18; hu24; hu31; hu34; hu43; hf2; hf4; hf5; hf19; hf20; hf21; hf22; hf28; hf33A; hf43; *H001; H591; H299; H383; H386; H420; H454; H605; H617; FVEC1302; FVEC1412; FVEC1456*	Y
**Animal (N = 52)**	dg97; dg99; dg100; dg101A; c67; c69; c70; c72; du77; du79, du80; du82; du83; du89; du112; du147; du149; du151; hs2; hs3; hs5; hs9; hs12; hs14; hs15A; hs16A; hs17; hs18; k2;; k3; k7; k8; k12; k126; k297;; *B921; B088; B367*: *B185; T426; TA143; MO56; TA447; TA144; TA255; TA271; TA054; M718; TA008; R424;*	Y
**Environmental/Unknown (N = 44)**	3A3; 4A2; 4A3; 4A4; 4A7; 4A10; 4A10_100; 4A13; 4B3_100; 5A1; 5A2; 5A4; 5A4_50; 5A5; 5A5_50; 5A6; 5A6_50; 5A7; 5A7_50; 5A8; 5A8_50; 5A9; 5A10; 5A11; 5A12; 5A13; 5A14; 5A15; 5A16; 5A17; 5A18; 5A19; 5A20; 5A21; 5B5A; 5B5B; 5B7_100; 5B14_100A; 5B14_100B; 5B16_100; *E560; E267; E1002; E1114*	Y
**Human (N = 59)**	hf1; hf3; hf6; hf8; hf9; hf10; hf12; hf14; hf15; hf16; hf17; hf18; hf23; hf24; hf30; hf31; hf32;; hf34; hu1; hu2; hu3; hu4; hu5; hu6; hu8; hu9; hu10; hu11; hu13; hu14; hu17; hu19; hu20; hu21; hu22; hu23; hu25; hu26; hu27; hu28; hu29; hu30; hu32; hu33; hu35; hu36; hu37; hu38; hu40; hu41; hu42; hu44; hu46; *H223; H588; H378; H413; H736*	N
**Animal (N = 27)**	dg90; dg92; dg93; dg95; c32; c33; c35; hs10; hs110; k4; k6; k9; k11; k15; du81; du84; du88; du103; *B367; TA249; TA280; TA464; M695; TA206; TA014; R527*	N
**Environmental/Unknown (N = 23)**	4A1_100; 4A2_100; 4A4_100; 4A5_100; 4A6; 4A8; 4B3; 4B5; 5A9_50; 5A2_50; 5A3_50; 5B1; 5B2_50; 5B3_15; 5B6_100; 4B7_100; 5B8_100; 5B9_100; 4B10_100; 5B18; 5B19_50; *E1118; E704*	N

^a^Isolate source codes:

Human isolates and strains: hu, human urine; hf, human faeces; H, human commensal (*in silico*); FVEC, human pathogen

Animal isolates: c, cattle; dg, dog; hs, horse; k, kangaroo; du, duck;

Animal *in silico* strains: B, bird; T, fish; TA, Northern Quoll, Native mouse, Bettong, Bandicoot, Potoroo, Tasmanian Devil; R, Reptile

Environmental samples (primary source unknown): Various combinations of numbers and letters (format eg. 4A5_100); E, Environmental.

### DNA extraction

Overnight broth culture of 500 μL was centrifuged at 10 000 × g for 1 min. Cell pellets were re-suspended in 180 μL DNase/RNase-free water and used for DNA extraction on the Corbett X-tractorGene automated DNA extraction system (Corbett Robotics, Australia). Phenol extraction for sequencing was performed manually according to the “OpenWetWare protocol” [26]. Briefly, cells were re-suspended in a TE buffer pH 8.0, incubated with RNase A (25mg mL^−1^) for 30 min at 65°C and then with proteinase K (25 mg mL^−1^) for 15 minutes at 37°C. After double extraction by phenol and chloroform, DNA was precipitated by ice-cold ethanol, and with 3M sodium acetate overnight with its final pellet washed with 70% ethanol and stored in DNase/RNase free water at −20°C until further use. The quantity and purity of DNA extracts were determined using a DU 730 spectrophotometer (Beckman Coulter, USA).

### Screening of *E*. *coli* strain collection for CRISPR 2.1

To screen isolates for the CRISPR 2.1 locus, primers 5′-TGGTGAAGGAGTTGGCGAAGG-3′ and 5′-AAAATGTCCCTCCGCGCTTACG-3′, annealing *iap* and *cas*2 respectively [13] were used with TaqPol (Roche, Australia) in a modified touch-down PCR reaction with the following conditions: denaturation at 95°C for 5 min, then 15 cycles, 95°C for 15 sec, 68°C for 1 min with decreasing temperature for each cycle, 72°C for 2 min; finally 20 cycles 95°C for 15 sec, 60°C for 1 min, 72°C for 2 min; final extension 72°C for 10 min. The amplicons were visualised using a 1% agarose gel, run at 100V for 30 min and stained using SYBR-Safe (Invitrogen, USA), and captured using the Gel-doc system (Bio-Rad, USA). CRISPR 2.1 presence was determined by the presence of appropriately sized bands (500–2000 bp).

### Cloning procedure and sequencing

Forty five isolates positive for CRISPR 2.1 loci were amplified using High Fidelity DNA polymerase (Bio-Rad, USA). The bands were excised from the gel, purified with QIAquick Kit (QIAGEN, USA) and ligated into a pGEM-3Z plasmid vector as described previously [27]. The plasmid DNA samples were submitted for sequencing to the Australian Genomic Research Facility, Brisbane. The sequences were annotated using ContigExpress, VectorNTI v.10 software (Invitrogen, USA) and were deposited in GenBank with accession numbers KF707494-KF707538.

### CRISPR analysis

CRISPR sets from sequences of CRISPR 2.1 locus were identified using the CRISPRFinder web database [7]. In addition, CRISPR loci from 54 *E*. *coli* genomes sourced from the Broad Institute (http://www.broadinstitute.org/annotation/genome/escherichia_antibiotic_resistance/GenomesIndex.htmL) were analysed *in silico* and 30 positive isolates carrying CRISPR loci were combined with our library.

### Direct screening of water samples and *E*. *coli* DNA for the known mobile genetic elements acquired from sequenced CRISPR arrays

Primers for the P7 phage were designed using Vector NTI (Invitrogen, USA) to target the corresponding spacer revealed from the *E*. *coli* CRISPR sequence analysis. A two litres water sample from the Brisbane River was filtered through a 0.33 μm filter. Genomic DNA (20 ng) from *E*. *coli* isolates were added as template for touch-down PCR reactions targeting 444 bp long amplicon of P7 bacteriophage by primer pairs (F: 5′-TCAAAATCCCCTGTTATCGT-3′ and R: 5′-TATTGTCTGAATGGTGGGGC-3′) with the following conditions: denaturation at 95°C for 5 min, then 15 cycles, 95°C for 15 sec, 65°C for 1 min with decreasing temperature for each cycle, 72°C for 2 min; finally 20 cycles 95°C for 15 sec, 60°C for 1 min, 72°C for 2 min; final extension 72°C for 10 min. Positive PCR products were excised from the gel and sequenced as previously described.

## Results

A set of 185 Australian *E*. *coli* isolates from different sources (animals, water and human) [24] was examined for the presence of CRISPR2.1 loci. Half of these were positive for the CRISPR 2.1 region (Table 1). Using the previously determined SNP profiles [24], 50 isolates were selected for sequencing from human and animal specific sources, and also mixed sources, which were positive for CRISPR 2.1. Isolates with identical SNP profiles and originated from the same plate and have similar/identical bands on the CRISPR 2.1 gel were excluded from current studies to minimize cloning and sequencing costs.

The sequences of these selected isolates and other Australian isolates downloaded from the Broad Institute website were analysed using CRISPRFinder, to detect direct repeats and spacers in these genomes. In order to sequence the CRISPR 2.1 regions, PCR products were cloned into pGEM-3Z vectors. Subsequently, the plasmid libraries were sequenced.

**Fig 1:**
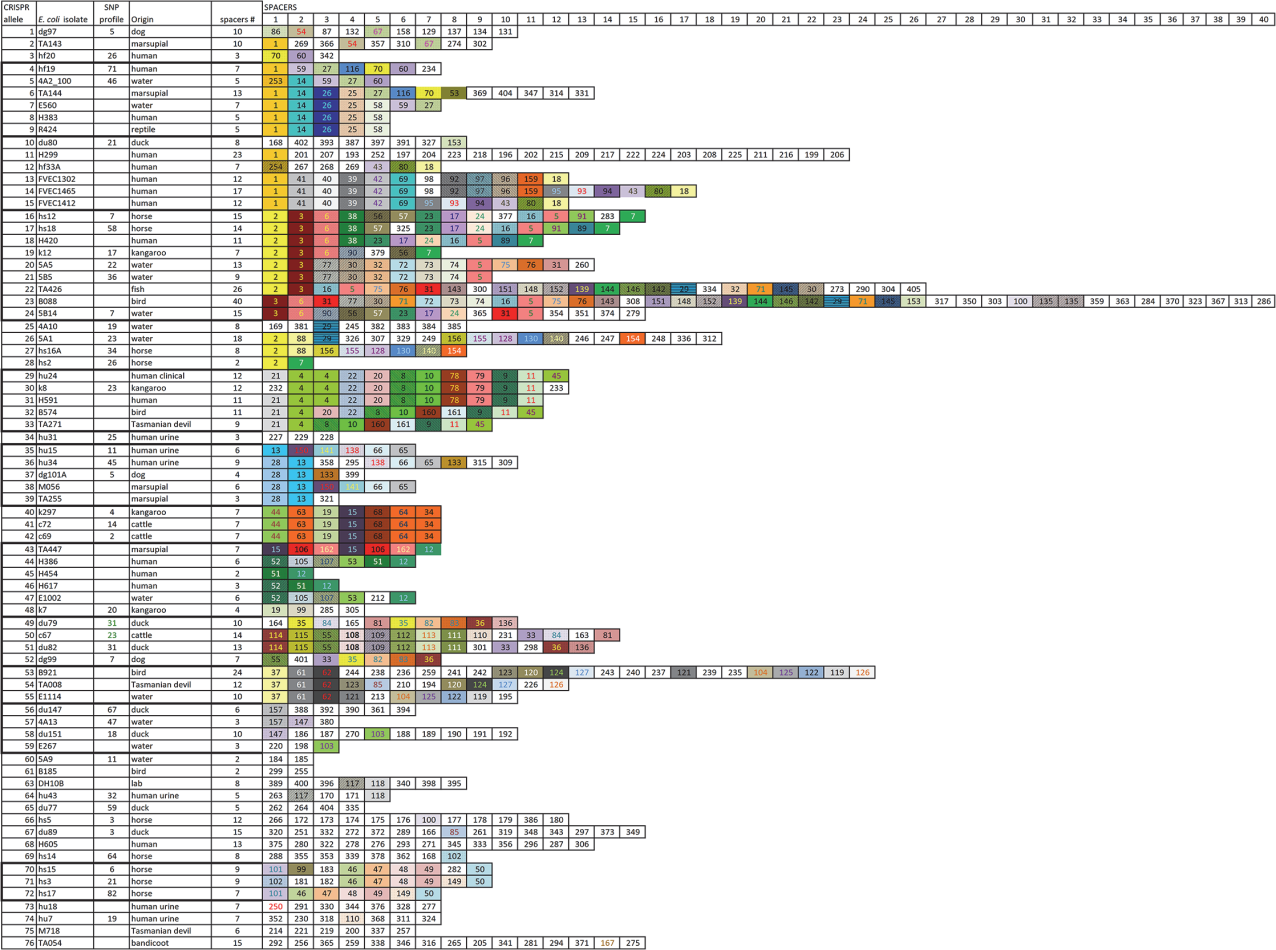
CRISPR allele types recorded from Australian *E*. *coli* isolates. Names of test isolates are written in lowercase and *in-silico* isolates in uppercase. Numbers and colors of spacers represent identical spacer sequences. Figure illustrates all spacer types found in this study, in chronological order from the oldest to the most recent, in position from left to right.

The spacers from all isolates tested and the aligned *in silico* strains, based on our library spacers’ sequences, are shown in Fig 1, together with their relevant SNP profiles. Each unique spacer array was assigned an allele number, and the primary source and SNP profiles were also included.

All spacer sequences were interrogated using the BLASTN algorithm in GenBank. This allowed for the extraction of specific proto-spacers which have been annotated thus far as mobile elements (plasmids, phages), in order to identify the influence of invading elements on *E*. *coli* diversity. The results of this BLASTN analysis, showing plasmids and phages, can be found in Table 2. This includes phage P7, *E*. *coli* plasmid pO111, pO157, amongst others.

**Table 2:** BlastN results of CRISPR 2.1 regions of isolates and *in silico E*. *coli*.

Isolates with same spacer	Source	Spacer (see Fig 1)	Proto-spacer			
			Phages	*Escherichia coli*	*Shigella*	*Salmonella*
Hf20, hf33H383, H299FVEC1302FVEC1412FVEC1465	Human	1	P7	*E*. *coli* O111:H plasmid pO111_2		
TA143, TA144	Tasmanian devil	1	P7	*E*. *coli* O111:H plasmid pO111_2		
R424	Reptile	1	P7	*E*. *coli* O111:H plasmid pO111_2		
Du80 4A2_100	Duck	1	P7	*E*. *coli* O111:H plasmid pO111_2		
E560	Water	1	P7	*E*. *coli* O111:H plasmid pO111_2		
Du151	Duck	4		*E*. *coli* O83:H1 str. NRG 857C plasmid pO83 *E*. *coli* ETEC H10407 p666 plasmid *E*. *coli* plasmid pEC_B24 *E*. *coli* 042 plasmid pAA *E*. *coli* O111:H- str. 11128 plasmid pO111_3 *E*. *coli* O103:H2 str. 12009 plasmid pO103 *E*. *coli* Vir68 plasmid pVir68 *E*. *coli* 0127:H6 E2348/69 plasmid pMAR2 *E*. *coli* 53638 plasmid p53638_75 *E*. *coli* 53638 plasmid p53638_226 *E*. *coli* 1540 plasmid pIP1206 *E*. *coli* SMS-3-5 plasmid pSMS35_130 *E*. *coli* O157:H- plasmid pSFO157 *E*. *coli* strain E2348/69 plasmid pMAR7 *E*. *coli* plasmid pAPEC-O2-R *E*. *coli* plasmid p1658/97 *E*. *coli* APEC O1 plasmid pAPEC-O1-ColBM *E*. *coli* plasmid pAPEC-O2-ColV	*S*. *flexneri* 2002017 plasmid pSFxv_1 *S*. *boydii* CDC 3083–94 plasmid pBS512_211 *S*. *flexneri* 2a str. 301 virulence plasmid pCP301 *S*. *flexneri* plasmid pINV_F6_M1382 TrbH *S*. *flexneri* virulence plasmid pWR100 *S*. *flexneri* 5a plasmid virulence plasmid pWR501 *S*. *flexneri* plasmid pSF5 *S*. *boydii* Sb227 plasmid pSB4_227 *S*. *dysenteriae* Sd197 plasmid pSD1_197 *S*. *sonnei* Ss046 plasmid pSS_046	*S*. *enterica subsp*. *enterica* serovar Kentucky pCS0010 *S*. *enterica subsp*. *enterica* serovar Kentucky pSSAP03302A *S*. *enterica subsp*. *enterica* serovar Kentucky pCVM29188_146
Hf20	Human	1		*E*. *coli* plasmid pO111		*S*. *typhi* R27 plasmid
Hf19	Human	5		*E*. *coli* plasmid pO111		*S*. *typhi* R27 plasmid
TA144	Tasmanian devil	7		*E*. *coli* plasmid pO111		*S*. *typhi* R27 plasmid
FVEC1302 FVEC1412 FVEC1465	Human	127	P7P1			
TA054	Tasmanian devil	814	TP Ogr (ogr)		*S*. *sonnei* plasmid pEG356	

Screening for the P7 phage sequences in filtered water showed an absence of free P7 phage DNA in the Brisbane River. In contrast, PCR amplicons of P7 genomes, using P7 phage specific primers, were identified in genomic DNA of *E*. *coli* hf19 which have spacers corresponding to the P7 phage and /or virulent plasmid from *E*. *coli* O111:H4.

## Discussion

### CRISPR diversity in human sourced *E*. *coli* isolates

Analysis of our mixed-source *E*. *coli* library revealed that CRISPR2.1 was present in some isolates from all the animals tested, however, the isolates from human sources tended to lack CRISPR 2.1 in general. About 75% of human isolates lack CRISPR 2.1 (53/71), in contrast to 30% (12/17) of *in silico* analysis of published Australian *E*. *coli* genomes. However, at least half of the *in silico* human-originating *E*. *coli* that lacked CRISPR 2.1 loci or where spacers were missing, were from the B2 phylogroup, which correlates with previously published data [21, 28] and low CRISPR presence in uropathogenic *E*. *coli* [29]. Indeed, the absence of CRISPR genes could be viewed as an indication of the presence of clinical and potentially pathogenic *E*. *coli* in water, as we found that the majority of our isolates from urine, blood and fecal clinical specimens also lacked CRISPR 2.1. Even if some strains could harbour CRISPR 2.1, the absence of the *cas2* gene may further support dysfunction of the CRISPR defence mechanism in clinical *E*. *coli* [30].

### CRISPR typing further resolves *E*. *coli* isolates with the same SNP and MLST profiles

We did not observe any consistent relationship between SNP profiles of our isolates or the Sequence Type (ST) of *in silico* strains and CRISPR alleles. Previously, evolutionary studies on CRISPR diversity of *Sulfolobus islandicus* demonstrated independent spacers’ acquisition in regards to genotype mutations in house-keeping genes [31]. This is due to the rapid CRISPR array evolution allowing different genotypes to acquire the same resistance to a common pool of viruses and plasmids [31], in contrast, house-keeping genes are more conserved.

Certain isolates with the same SNP profile were found to have different CRISPR alleles. For instance, SNP profile 23 comprises isolate profiles of kangaroo (k8); cattle (c67) and a water sample (5A2). These three isolates had completely different CRISPR alleles (CA) confirming that the three isolates were indeed from different sources, despite their common SNP profile. Since one of the aims of this project was to further discriminate between *E*. *coli* isolates with common SNP profiles, and in this instance, we have proven this is true, however it is obvious that further work is required to discern whether more mixed-source SNP profiles may be discriminated using this approach.

Another type of relationship was observed in ST10, a genotype common to three human *E*. *coli* isolates (H386, H454, H617), and one from water (E1002). These isolates had similar but not identical spacer sequences. However, the pattern was not followed with human isolate H383 (also ST10), which has a different CRISPR allele compared to the other ST10 isolates. Therefore, the use of CRISPR diversity may allow distinction between isolates from different host origins, which were previously combined in one ST and consequently in one SNP profile [24].

### Identical *E*. *coli* CRISPR arrays indicate that isolates could be from the same geographical location

In contrast, other isolates had different SNP profiles but the same CRISPR allele pattern. For instance, *E*. *coli* isolate c69 originating from cattle- (SNP profile 2) had the same CRISPR allele (CA 40–42) as cattle isolate c72 (SNP profile 14) and kangaroo-originating k297 (SNP profile 4). The only identical spacers to be found in *E*. *coli* were from these three animal faecal samples originating from one farm site. As the likelihood of identical CRISPR 2.1 alleles in unrelated organisms is very low, we postulated that it was likely that these three isolates were identical because they were from the same geographical location. Since CRISPR spacer arrays, which are undergoing rapid horizontal gene transfer events, can change rapidly within a few generations of *E*. *coli* growth in a host, it is very probable that only those hosts sharing a food source, and in close physical proximity to each other, will have identical CRISPR spacer arrays. However, recent studies reported the conservative character of CRISPR alleles [28, 32]. According to these studies, *E*. *coli* strains which diverged about 250 000 years ago have identical CRISPR arrays compared to modern strains, indicating a surprisingly low level of diversity. Nevertheless, our CRISPR library shows a high diversity of spacers from a wide range of hosts and uniquely similar arrays only within those *E*. *coli* isolates which were sampled from one geographical location and at one time.

Identical alleles were also observed from *in silico* animal and human *E*. *coli* sequences: isolate R424 (ST34) from a common suburban garden skink and human *E*. *coli* H383 (ST10) (Fig 1). Another example of matching CRISPR alleles was observed in the group CA6-9, which combined human, reptile and medium-sized carnivorous marsupial *E*. *coli* isolates, as well as isolates from water. While the host source of these isolates is known, the geographical area in which the hosts were contained was not supplied, so it was not possible to verify the explanation tendered above.

### Proto-spacers identified in Australian *E*. *coli* isolates

This study established that the enterobacterial P7 phage proto-spacer in *E*. *coli* was found in Australian animal and human isolates and also in isolates from environmental water sources. Mostly this proto-spacer was found in association with plasmid pO111_2, normally found in *E*. *coli* O111 EHEC (Table 2).

Previously, proto-spacers of P1 phage and F plasmid have been found in *E*. *coli* genotypes ECOR44 and 47 and ECOR 42 and 49 respectively [13]. This led to further analysis of our data in order to find the link between DNA phages/plasmids and proto-spacers in Australian *E*. *coli* isolates.

Of particular interest was the large number of isolates that had the proto-spacers P7 and pO111_2 [33]. This showed that these plasmids carry virulence genes which change non-virulent O157 *E*. *coli* into pathogenic O157 ETEC strains. Taking into account the large proportion of human isolates with spacers against P7/pO111, it can be seen that similar past attack events seemed to have occurred in many of the isolates analyzed here. The observed insertion of the proto-spacer pO111 into the genome of past isolates may indicate the frequent interaction with genetic mobile elements.

Such strains either become resistant to the conjugative plasmid pO111 or become repressed by CRISPR immunity, if this plasmid is acquired. Resistance to plasmids was recently reported in *Staphylococcus aureus* livestock ST398-MRSA-V strains, which explains why such strains have less antimicrobial drug-resistant genes and phage-encoded virulence factors compared to other MRSA strains [34].

### Proto-spacers and CRISPR immunity

Extensive studies into the molecular mechanism of CRISPR immunity have shown that insertion of proto-spacers into the CRISPR array occurs in the next position after the first direct repeat, which locates them in close proximity to the leader sequence. Thus, spacers are stored strictly in chronological order with the oldest at the end (spacer 1, see Fig 1). The most intriguing matches were shown for spacer 270 of the duck isolate du151. *In silico* predictions revealed a potential linkage between this spacer and plasmids carrying virulence genes (Table 2). Interestingly, spacer 270 was not only predicted to target *E*. *coli* plasmids, but also plasmids of other enterobacteria such as *Shigella* and *Salmonella*. For instance, spacer 270 targets plasmid p666, which confers ETEC pathogenicity to *E*. *coli* H10407 [35]. Another plasmid pO83 (150 kB in length) [36], carries adhesive factors, which allow *E*. *coli* to become adherent and invasive *E*. *coli* (AIEC), which is associated with Crohn's Disease. This plasmid has >85% identity with two other plasmids, pAPEC-01-ColBM and avian pathogenic *E*. *coli* (APEC), and also with the plasmid pCVM29188_146 of *Salmonella enterica* serovar Kentucky [37]. These plasmids share the common function of colicin M and D production. The same proto-spacer sequences were present in the large plasmid pEC_24 with 73.8Kb which was previously identified from a number of clinical isolates [38]. Interestingly, as well as carrying multiple-antibiotic resistance genes, this plasmid also harbours genes responsible for colicin production that Smet and co-authors had found never to have been reported before for such IncFII class [38]. *E*. *coli* uses these colicin toxins to compete with other strains of the same species, as acquisition of these plasmids can give an advantage to the host by killing the strains which do not produce colicins [38]. This may lead to the explanation of why the spacer was not commonly identified in the *E*. *coli* population. Particular duck *E*. *coli* isolate 240, which has a distinctive allele and could have an ecological niche. This isolate probably does not require colicin production due to lack of interaction with *E*. *coli* strains in the gut. Further detailed studies are needed to prove this assumption.

As noted earlier, the CRISPR system targets the most vital genes coding for key proteins in the replication process of the mobile elements’ replication, or the conjugation process [13, 39]. Using *in silico* predictions, we found that du151 spacer 270 targets: OriT nicking and unwinding protein, a type IV secretion-like conjugative transfer system pilin acetylase TraX of the *Shigella* species; a relaxase protein TraI of the same family in *Salmonella typhi* plasmid, and conjugal transfer nickase/helicase TraI in the *E*. *coli* F plasmid. Indeed, CRISPR interference with plasmids was initially discovered on plasmid nickase genes of *Staphylococcus epidermidis* [40] which is vital for self-replication. Thus, current results are further evidence of the universal nature of the CRISPR resistance mechanism in *E*. *coli*.

Interestingly, high numbers of old acquired spacers matched sequences that were flanked by aminopeptidases and some non-annotated conservative protein genes, from CRISPR sites of *E*. *coli* serotypes O111:H-, O113:H2 and O26:H11. Isolates from different sources had such spacers, which were, however, different from known mobile elements. This finding poses a question about the existence of such mobile elements that might not yet have been isolated. Alternatively, it may be that such elements have disappeared or have become degenerated, and can now be found only as a part of the bacterial genome. Such spacers were identified in isolates from horses and water samples only, so they are poorly studied. These isolates, however, are identical to CRISPR arrays of pathogenic strains of *E*. *coli* in GenBank, suggesting that they are likely to have shared the same pool of mobile elements in the past. Future studies on host-virus interactions could reveal such conjugative plasmids and phages that remain unknown at present.

### Conclusion

It was found in this study that CRISPR array analysis alone was not effective as a source tracking tool for *E*. *coli*, which led us to intensify the search for unknown phage sequences. The high diversity in *E*. *coli* CRISPR can be an advantage when the level of specificity is required to be high (for instance, in the case of proof of site identity). Such a tool may involve the combination of SNP genotyping and CRISPR allele identification based on a high resolution melt approach. The fact that isolates from two sources shared an allelic profile out of 50 isolates analyzed, led us to apply the preliminary results using a combination of methods for microbial source tracking. CRISPR spacers, harbouring sequences of phage and plasmids, may prove useful in investigating host-specificity of these invading elements. Analysis of *E*. *coli* CRISPR patterns showed a lack of host-specificity for all isolates sequenced in this study.
